# Impact of Overtraining on Cognitive Function in Endurance Athletes: A Systematic Review

**DOI:** 10.1186/s40798-023-00614-3

**Published:** 2023-08-08

**Authors:** Isabella K. Symons, Lyndell Bruce, Luana C. Main

**Affiliations:** 1 School of Exercise and Nutrition Sciences, Faculty of Health, Deakin University, 221 Burwood Highway, Burwood, VIC 3125 Australia; 2 Centre for Sport Research, Deakin University, Geelong, Australia; 3 Institute of Physical Activity and Nutrition, Deakin University, Geelong, Australia

**Keywords:** Overload, Overreaching, Athlete monitoring, Cognitive performance

## Abstract

**Background:**

Endurance sports require significant training loads to elicit the desired training effects on an athlete’s body. However, if adequate recovery is not provided, overtraining may occur, with corresponding decrements in performance. As such, there is a need for measures that can be tracked, to monitor athlete adaptation to training loads, and provide early warning of possible maladaptation. The aim of this review was to determine if a relationship exists between overtraining and cognitive function in endurance athletes.

**Methods:**

A systematic search of AMED, MEDLINE, SPORTDiscus and APA PsycINFO was conducted. Eligibility criteria included original peer reviewed research, written in English, published between January 2000 and May 2022, and using human participants. Of the 221 articles screened, a total of seven studies met the inclusion criteria and were included in this review.

**Results:**

The findings of the review suggest that there is a relationship between overtraining and cognitive function with all seven studies finding that cognitive performance declined in response to athletes becoming overreached or overtrained. These studies found that reaction time (as measured by Stroop colour test) increased in response to both overreaching and overtraining.

**Conclusions:**

Cognitive function tests such as the Stroop Colour Test could be included as part of a broader programme for monitoring athlete adaptation to training.

## Key Points

- Cognitive function was impaired in athletes who were overreaching or overtraining.
- Reaction time appeared to increase with overtraining.
- Future research should examine whether tests of cognitive function could be used as part of routine athlete monitoring programs to identify early signs of non-functional overreaching.

## Background

Coaches often overload athletes to enhance their performance by challenging the body to effect a supercompensation response [1]. Consequently, this leads to athletes feeling fatigue during and after training sessions. In particular, endurance sports require significant training loads to elicit the desired training effects on an athlete’s body. To achieve the required training stimulus, training is periodised around the competition season, and will include short periods of overload, sometimes referred to as ‘overtraining’ (i.e. to train more or harder) followed by periods of recovery. These periods elicit a deliberate short-term training state of functional overreaching (FOR), where sports performance will temporarily decrease in response to training. However, with adequate recovery, positive physiological adaptation will occur, with a commensurate improvement in performance capacity [2, 3]. In contrast, with prolonged periods of intensive training, or where adequate recovery is not provided, non-functional overreaching (NFOR) may occur, which left unchecked may ultimately develop into overtraining syndrome (OTS) [4]. This undesirable training state (i.e. NFOR) represents the accumulation of training and non-training stress which lead to negative physiological changes and a decrement in performance. Depending on severity, performance capacity may take several weeks to months to return to baseline performance state [4]. Overtraining syndrome, however, is more severe than NFOR, and occurs when the accumulation of training and or non-training related stress results in a long-term decrease in performance and is often accompanied by other physiological and psychological fatigue or illnesses [3, 4]. This maladaptive response to the training load can require several months to years for full recovery [4].

Therefore, there is a need for measures that can be used to track and manage athlete adaptation to training loads and provide early insights into the possible risk of an adverse event or training response occurring. Currently, differentiation between these training states (FOR/NFOR/OTS) cannot be diagnosed using any one tool but rather is based upon either the time course for recovery, and or the exclusion of other factors which may have led to this decrease in performance [3, 4]. Decrements in performance require physical performance tests to monitor (mal)adaptation or recovery, but these are often undesirable as they only serve to further overload the athlete. Although a range of objective measures have been examined [5], many of these require access to expensive resources or laboratory equipment that are beyond most sporting clubs and organisations. As such, measures of subjective wellbeing are more often used to monitor athlete responses to training [5]. However, these are not without their limitations, and there is always a risk that athletes will under-report symptoms of training distress if they fear negative repercussions (i.e. de-selection). Therefore, there is a need for objective measures that can be tracked to manage athlete adaptation to training loads and provide early insights into risk of a maladaptive response. Currently there is no reliable, low burden objective measure current state or prospective performance capacity available for coaches and support staff to monitor athlete adaptation to training.

The association between cognitive function and endurance training is not fully understood; however, some research has attempted to examine this relationship. Cognitive function refers to the mental abilities of a person including learning, thinking, reasoning, remembering, problem solving, decision making and attention [6]. There is a recognised association between exercise and improvements in cognitive function [7, 8]. However, physical stress, be it from pressures put on the body during exercise, or other physical stressors, and psychological stress associated with pressure from work or school, has been associated with a decline in cognitive function [8]. Impaired cognitive function can cause memory loss, difficulties paying attention as well as increases in reaction time which may be associated with an increased risk of injury [7]. Theoretically, during periods of excessive physical stress imposed by training overload, a decrement in cognitive function would be observed due to the accumulation of stressors on the body. However, the majority of research thus far has focussed on short-term cognitive function decline based upon the fatigue during a single exercise session.

To date, normal levels of fatigue experienced during standard participation in sport competition have not been shown to impact decision making [9]. However, when athletes reach levels approaching exhaustion, decision making has been shown to decline [9]. This accumulation of stressors across physical and psychological domains of the athlete may inherently increase cognitive impairment in athletes. Therefore, increased stress and consequent impaired cognitive function may be observed in endurance athletes who experience FOR/NFOR or OTS. The aim of this review was to systematically examine the current literature to determine if there was any evidence of a relationship between FOR/NFOR and OTS, and cognitive function in endurance athletes, and to investigate the possibility of using cognitive tests to detect onset of FOR/NFOR.

## Methods

### Search Strategy

This systematic review was developed in adherence to the guidelines of the Preferred Reporting Items for Systematic Reviews and Meta-analysis (PRISMA). The PRISMA checklist is used as the basis for reporting systematic reviews [10]. The review protocol was not pre-registered for this review. A literature search was conducted in May 2022 using the EBSCOHost search engine and the electronic databases of AMED, MEDLINE, SPORTDiscus and APA PsycINFO. The literature search was restricted to papers written in English, human studies and those published between January 2000 and May 2022. The search strategy used is presented in Table 1. This initial search yielded 221 results with an additional 6 studies found through a manual search.

**Table 1:** Database search strategy

Overtraining	overtrained OR overtraining OR overtrain* OR staleness OR "performance plateau" OR "overtraining syndrome" OR "over reaching" OR "poor recovery" OR "under recovery" OR "over train" OR "over-train" OR "over trained" OR "over-trained" OR "over training syndrome" OR "over-training syndrome"
Cognitive function	“cognitive function" OR cognition OR perception OR memory OR learning OR "decision making" OR "mental abilities" OR "brain function*" OR "working memory" OR memory OR "cognitive flexibility OR "mental operations" OR academic OR "academic success" OR thought OR "though processes" OR intellect* OR conscious* OR "psychological function" OR judgement OR attention OR *conscious* OR mind OR recollection OR remembrance
Endurance athlete	athlete* OR "sports person*" OR "sports people" OR sportsperson OR sportspeople OR sportsm?n OR sportswom?n OR "sports m?n" OR "sports wom?n" OR run* OR swim* or sail* OR triathlon OR triathlete OR row* OR "cross country skiing" OR "physical training" OR "physical activity" OR "endurance training" OR "endurance sport*" OR "endurance athlete*" OR "open water swim*" OR "water polo" OR "ultra marathon" OR football OR soccer OR "endurance training" OR marathon OR “badminton player*” OR baseballer* OR “baseball player*” OR basketballer* OR “basketball player* OR bastm?n OR bobsledder* OR *boxer* OR canoeist* OR cricketer* OR *cyclist* OR “hockey player*” OR kayaker* OR “lacrosse player*” OR *marathoner* OR netballer* OR “netball player*” OR racewalker* OR “race walker*” OR softballer* OR “softball player*” OR “tennis player*” OR volleyballer* OR “volleyball player*” OR aerobic OR "aerobic type" OR "endurance type" OR "aerobic athlete*" OR "aerobic sport" OR sport OR sporting OR exercise

### Study Selection

Search results were initially screened by title and abstract against the selection criteria by the first author (IS). Full text screening was then undertaken on the included results by the first (IS) and third (LM) authors. The selection criteria for the review were as follows.

#### Inclusion Criteria

1. Study was peer-reviewed and original research.
2. Participants were from an athlete population.
3. The study explored effects of overtraining on cognitive function.

#### Exclusion Criteria

1. Research written in languages other than English.
2. Studies on population groups other than athletes.
3. Studies with subjects which were not human.
4. Systematic or literature reviews.
5. Studies on mental health disorders.

### Data Extraction

Data were extracted from all included studies into a table by the first author (IS). Data which were extracted included the year the paper was published, population demographics, overload protocol, cognitive measure/s used as well as outcomes and likely training state based on the European College of Sport Science (ECSS) consensus statement [4].

### Quality Assessment

As no suitable published assessment criteria were available each study was assessed for quality using a modified version of the Newcastle–Ottawa Scale [11]. This assessed participants, measurement tools and outcome of the study. Each section was scored out of a possible 2 with the highest possible cumulative score being 14 out of 14. The cumulative score was used to provide an overall assessment of quality of evidence. Scores greater than 11 were considered high quality and scores 8–11 were considered moderate quality.

### Risk of Bias Assessment

Studies were assessed for risk of bias through giving a weight to their contribution to the study. Each criterion (except for criterion 1.) was scored out of 2 with the highest possible cumulative score for each study being 9 (see Table 2). Studies with a risk bias assessment score of 8 to 9 were considered a low risk of bias, whilst studies with a scores between 4 and 8 were considered as having a moderate risk of bias [5]. Scores of 4 or less had a high risk of bias [5].

**Table 2:** Risk of bias assessment criteria

Criteria	Definition	Scoring		
		0	1	2
1. Peer reviewed	Study published in peer-reviewed journal	No	Yes	
2. Number of participants	Number of participants included in study findings	< 5	5–50	> 50
3. Population defined	Age, biological sex, sport, participation level and experience stated	No	Partly	Yes
4. Training load described	Training or competition load described as overtrained or overreaching	No	Overreaching	Overtrained
5. Measure of cognitive function described	Measure of cognitive function described (e.g. Stroop test, response time test)	No	Partly	Yes

## Results

**Fig. 1:**
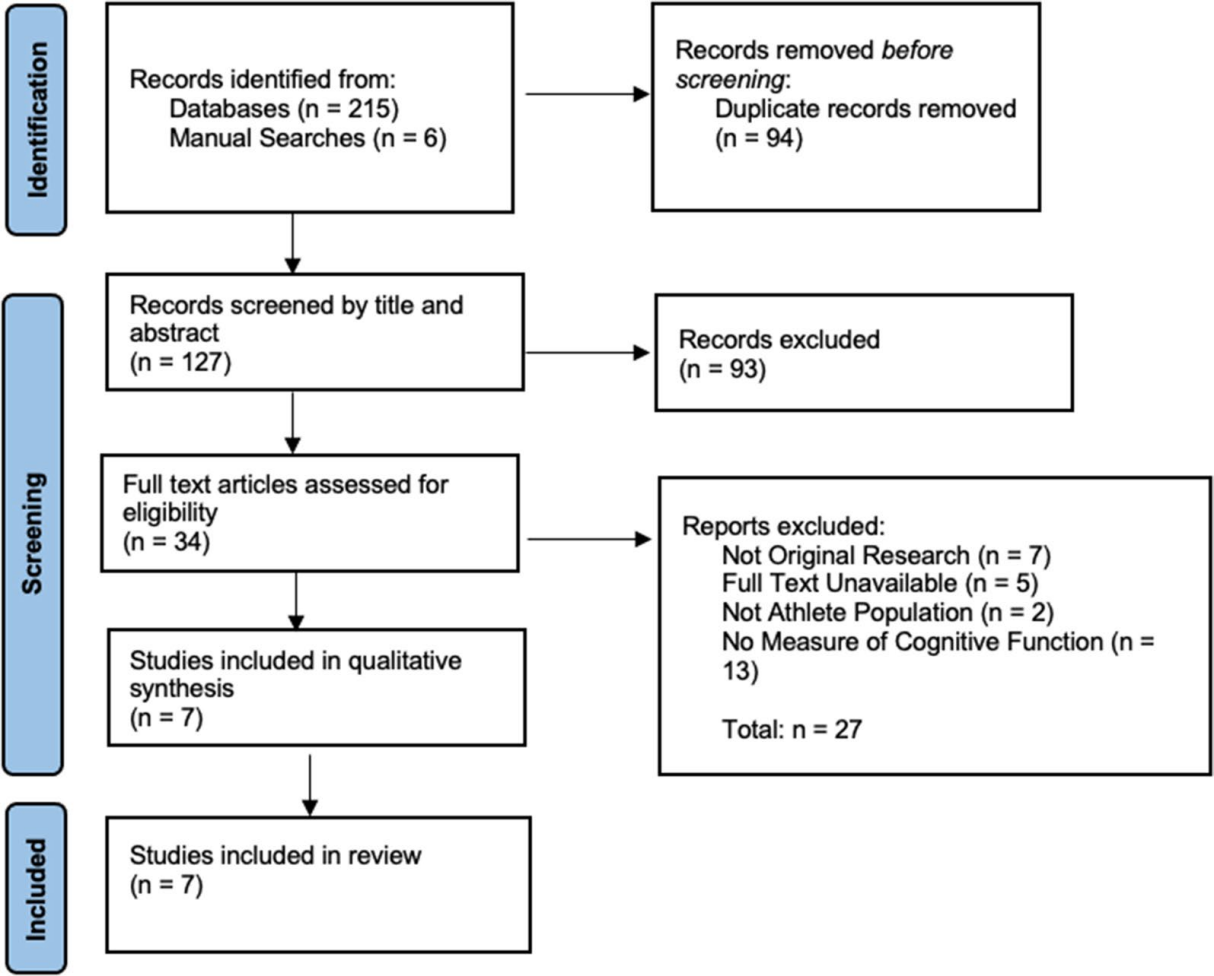
Screening process for selection of studies in relation to inclusion and exclusion criteria

After removal of duplicates, 127 article titles and abstracts were screened for relevance using Covidence [12]. After this screening, a further 93 articles were removed. Thirty-four articles remained for full text screening. During full text screening, another 27 articles were excluded for not meeting the inclusion criteria, or because one or more of the criteria for exclusion were met. Seven articles met all criteria for inclusion in the current review. The screening process is described in Fig. 1.

The average quality of evidence included in this study was moderate with 43% of evidence considered high quality and 57% of evidence considered moderate. No evidence was of low quality. Five of the seven studies scored a risk bias of 7 and two posed a risk bias of 6 this, resulting in an average risk bias of 7 meaning that there was a moderate risk of bias (see Table 3).

**Table 3:** Risk of bias assessment

Study	1. Peer reviewed	2. Number of participants	3. Population defined	4. Training load described	5. Measure of cognitive function described	Total
Blain et al. [14]	1	1	2	1	1	6
Dupuy et al. [15]	1	1	2	1	2	7
Dupuy et al. [16]	1	1	2	1	2	7
Hynynen et al. [13]	1	1	1	1	2	6
Le Meur et al. [18]	1	1	2	1	2	7
Nederhof et al. [19]	1	1	2	1	2	7
Rietjens et al. [17]	1	1	2	1	2	7

The participants examined in the studies were reasonably homogenous, with all participants endurance trained in either triathlon, swimming, running, or cycling. The mean age of the athletes across all studies was 28 + 4.8 (range 24 ± 3 years to 36 ± 1.5 years). Only one study included a group of athletes who were in a state of OTS [13]. Five studies contained athletes who had achieved FOR [14, 15]. Periods of overtraining and the way in which studies measured athlete training load varied. Studies included in the review spanned from 2 to 9 weeks, with three of the studies using an overload period of 2 weeks[15–17], and two using a 3-week overload period [14, 18]. One study measured cognitive function after a 10-day training camp [19], and one measured cognitive function and cardiac autonomic response within 6 weeks of being medically diagnosed as suffering from OTS [13]. Just over half of the studies (*n* = 4) included a control group (i.e. not overtraining).

Five studies investigated the impact of an overload training protocol on cognitive function where athletes overtrained, whilst one specifically investigated clinically diagnosed overtrained athletes (OTS). In six of the manuscripts reviewed, athletes were referred to as ‘overreaching’. However, in five of these studies, participants most likely reached a FOR state, given the group mean improvements in performance following a recovery period [14–16, 18, 19]. In one manuscript [17], it was not possible to determine likely training state (i.e. FOR or NFOR) as data collection appeared to cease at the end of the overtraining period, and there were no follow-up measures taken after the 1-week recovery period.

Like training load, different measures of cognitive function were included in the studies reviewed. These tests ranged from decision making tasks, to memory and learning, and simple vigilance or reaction time tests. In the seven studies included, three studies used the Stroop colour word test, four used a variation of a cognitive reaction time test, one used behavioural tasks, and one study used psychomotor speed to determine the effect training load has on cognitive processes. Table 4 provides a summary of each of the included studies.

**Table 4:** Summary of evidence of the seven articles included in the review

Study	Population sample	Overload protocol	Cognitive measure/s	Outcomes	Likely training state based on ECSS consensus
Blain et al. [14]	37 male triathletes: *n* = 19 FOR (35 ± 1.2 years) *n* = 18 control (36 ± 1.5 years)	8-week study design with 3-week overload protocol (40% ↑ in training load)	Behavioural choice task (i.e. subjective preference) and a cognitive task (i.e. N-Back/N-Switch)	Training induced fatigue ↑ the attraction of immediate versus delayed rewards in economic choice task; but not the way value options were estimated and compared. No sig. differences between groups in cognitive task performance	FOR
Dupuy et al. [15]	10 male endurance athletes (31 ± 6 years): *n* = 6 road running *n* = 2 road cycling *n* = 2 triathletes	2-week overload period (100% ↑ from baseline training volume) 5 athletes were assigned to the negative adaptation group (NAG) following overload training	Simple reaction time task and a computerised version of the Stroop colour-word test	No sig. differences between groups in Stroop task performance. Effect size show small ↑ in NAG and a moderate ↓ in PAG in reaction time. Error rate did not change in congruent, denomination or interference tasks. For switching task ↓ after overload training in NAG, suggesting a ↓ in performance that required the use of executive function with FOR Sig. group × time interaction for simple reaction time, with initiation time significantly slower for NAG following overload	FOR
Dupuy et al. [16]	11 male endurance athletes (29.5 ± 9.3 years): *n* = 6 road running *n* = 2 road cycling *n* = 3 triathletes	2-week overload period (100% ↑ from baseline training volume)	Computerised modified Stroop Test	Slowing of cognitive performance following 2-week overload period. Moderate ↑ in overall RT to Stroop tasks after overload (*p* = 0.03) returned to base after 1 week taper, with small/moderate ↑ in RT for denomination (*p* = 0.04) and interference conditions (*p* = 0.01) after overload. NS tendency for increase in FT with switching condition (*p* = 0.07)	FOR
Hynynen et al. [13]	24 endurance athletes: *n* = 12 Control *n* = 12 overreaching Both groups 50:50 male: female split	Clinically diagnosed as OTS versus control group	Stroop colour word test	Overtrained athletes made sig. more errors than control, suggesting signs of ↓ cognitive performance when overtrained compared to a normal training state. OTS athletes performed poorer than control when time to respond to stroop task was reduced (moderate, *p* = 0.046; and fast speeds, *p* = 0.045). No difference (similar) in slow speed	OTS
Le Meur et al. [18]	24 triathletes: *n* = 8 normal training *n* = 16 intense trained (IT) Sex not reported	IT group completed a 3-week intensified programme designed to deliberately overtrain the triathletes; Duration of each training session of the classic training period was ↑ by 40% *Only 11 of 16 were truly OR	Audio stimulus reaction time test	No difference in cognitive performance at low intensity function between control group and overtraining group; however, at exhaustion there was a sig. ↓ in cognitive performance OR group showed sig. ↓ in performance (more false responses to audio stimulus task) only at exhaustion (not at rest, low intensity, or lactate threshold) relative to normal training group	FOR
Nederhof et al. [19]	28 cyclists: *n* = 14 control (9 male; 5 female) *n* = 14 overreaching (10 male; 4 female) of which: *n* = 7 well trained; *n* = 5 FOR; *n* = 2 excluded from analysis	The high load training period consisted of a regular training camp during which the cyclists performed their own training schedule. The training camp lasted on average 9.5 days (SD = 2.8). This was for the 14 well trained cyclists	Psychomotor speed: finger pre-cueing task Determination test	No sig. differences in psychomotor speed between groups on finger pre-cueing task; trend for reduced psychomotor speed in FOR athletes after training overload versus control. Again, on determination task, trend for delayed in reaction time for the two faster presentation intervals for participants after the training camp group versus control. No group differences for action part of determination test; no group differences for reaction times of determination test	FOR
Rietjens et al. [17]	14 male cyclists *n* = 7 experimental group (25.3 ± 4.7 years) *n* = 7 age matched control group	2-week intensified training period, preceded by 2-week pre-intervention baseline training period	Video stimuli reaction time test	The most sensitive parameter for detecting overtraining was reaction time	FOR/NFOR unable to distinguish as no measures after recovery period

↑ = increase, ↓ = decrease, −ve = negative, Sig. = significant/significance

### Stroop Colour Word Test

Three of the papers reviewed included the Stroop colour word test. This test is used to evaluate overall executive function and measures a person’s cognitive flexibility, selective attention capacity and processing speed. It involves four different conditions, the first involving identifying colour names written in their corresponding colour (i.e. red, green, blue or black) [13, 15, 16]. The second condition involves repeating the task but identifying the colour name written in a different colour than the word [13, 15, 16]. The third condition involves naming the colour of a word not the word itself (e.g. RED written in green) whilst the fourth condition is the same but includes a square appearing in 25% of trials meaning the colour of the word not the word has to be identified [13, 15, 16]. In all trials, reaction time and error rates are recorded. From the manuscripts reviewed, reaction time during the Stroop test increased in athletes that were in a state of FOR [15, 16], or athletes who had been diagnosed as overtrained [13]. The number of mistakes made or the error rates also increased as training overload increased or was present, although this was only observed in the switching task [15]. This was particularly noticeable in the studies which undertook tests at moderate to high speeds, with these studies not finding a significant difference at low speeds when compared to control or groups who were training overloaded [13, 15, 16].

### Reaction Time Tests

Reaction time tasks require participants to react to either a visual or audio stimulus occurring at random intervals, and responding by pressing a button [17, 18]. Four of the studies included in the review used reaction time tests or speed task tests as a measure of cognitive function [15, 17–19]. All studies found reaction time to be compromised following a period of overtraining in athletes that were likely in a FOR/NFOR training state. Significant differences for reaction time were found in three of the studies with athletes presenting as overreached having slower reaction times [15, 18, 19]. Notably, although FOR athletes had slower reaction times at rest and after low intensity exercise, no significant differences were found when compared to control groups [18]. It was not possible to determine whether the athletes in the Rietjens et al. [17] were FOR/NFOR due to the absence of measures following a recovery period. Regardless, the most sensitive parameter for detecting overtraining was reaction time [17].

### Behavioural Choice Tasks

The behavioural choice task used involved participants indicating a preference for immediate rewards or bigger-later rewards. Training induced fatigue increased the attraction of immediate vs. delayed rewards in economic choice task; but not the way value options were estimated and compared [14]. The study using behavioural choice tasks to examine the effects of overreaching on cognitive function compared overreached athletes to a control group who did not undertake the overreaching protocol [14]. It was found that the athletes who were overreaching displayed a higher proportion of impulsive choices and increased the attraction of immediate rewards [14]. In contrast the control group did not appear to be as impulsive or demonstrate as high immediacy bias [14].

### N-Back test

If a letter presented was the same as was presented *n* trials ago or different, with difficulty manipulated from *n* = 1 before to *n* = 3 before. In the second task participants had to determine if the letter was a vowel (a, e, i, o, u) or consonant (b, c, k, m, p) when the letter appeared green or if the letter was upper or lower case when the letter appeared red [14]. There were no significant differences between groups on the task performance, suggesting that although the athletes were fatigued, the physical exhaustion was insufficient to affect cognitive control [14].

### Psychomotor Speed

The psychomotor speed test used was a finger pre-cueing test which used plus signs presented as stimuli consisting of a warning signal, a cue signal, and a target signal [19]. Responses were given by pressing keys using the index and middle fingers of both hands. The aim was to respond to the stimulus as quickly as possible by pressing the appropriate keys. The study which used a pre-cueing task to measure psychomotor speed investigated the effects of high training loads on psychomotor speed [19]. It found that after high training loads reaction time to the task was slower.

## Discussion

This review sought to explore the effect that FOR/NFOR/OTS has on cognitive function in endurance trained athletes. The review found that excessive increases in training load (i.e. *overtraining*) negatively influenced cognitive function in athletes. Reaction time increased in both reaction time tests and Stroop colour word tests; psychomotor speed decreased, indicating compromised performance. These declines in cognitive processing were due to a prescribed overload training block or athletes being in a clinically diagnosed overtrained state (OTS). These studies also found that there was an increase in impulsivity in behavioural tasks, with overloaded athletes having a higher proportion of impulsive choices.

Although only one study included athletes that were clinically suffering from OTS, all studies showed similar trends, with athletes who were in a state of functional to non-functional overreaching showing signs of impaired cognitive function. A clinically diagnosed OTS athlete population was assessed using the Stroop colour word test and time to respond to the Stroop task was reduced; athletes who were overtrained made significantly more mistakes than the control athletes [13]. This finding was similar to the overreaching studies which included the Stroop colour word test. The functionally overreached athlete populations athletes made more mistakes and had slower reaction times [15, 16]. Studies which included reaction time tasks as a measure of cognitive function in overreached and functionally overreached athletes were similar; as fatigue increased, the athletes’ reaction time increased [15, 17–19]. There was consistency across all studies showing that cognitive performance, irrespective of the measure, decreased as athletes became overreached or overtrained. This suggests that there is a relationship between overtraining and cognitive function.

Being able to detect athletes at potential risk of NFOR or even OTS may be beneficial so periods of recovery can be implemented before OTS and performance decrements occur. The potential magnitude of the sporting performance decrement is large, as reaction times can be up to 20% slower in overreached athletes compared to a control group [19]. This is despite motor control not appearing to decline in response to FOR [18]. It is likely that athletes who are experiencing fatigue from training overload will be able to continue training; however, information processing and decision making may become impaired, particularly if the training overload and fatigue continues.

The Stroop colour test which allows for the measurement of cognitive function such as attention, processing speed and cognitive flexibility [20] was able to identify differences between athletes who were overtrained and those who were not. Thus, this test could be considered as another monitoring tool to manage the health and wellbeing of athletes, providing an early indication of a possible decline in cognitive function as a consequence of training overload. As cognitive function appears to be a sensitive parameter for potential overtraining in athletes, the Stroop test could be used as an early detection tool and potentially allow for adjustments to training before an athlete progresses further along the overtraining continuum. Similar results have also been observed in other physically demanding contexts, including a military population. During physically demanding military operations, decrements to cognitive performance were greater than decrements to physical function [13].

Studies which explored the effect of overreaching on cognitive performance found that cognitive function as measured by reaction time was able to return to baseline after a short period (i.e. 1–2 weeks) of recovery in athletes who were in a state of functional overreaching [16, 19]. Athletes who had accumulated greater levels of fatigue were found to require longer than a week of recovery time and were often classified as undergoing non-functional overreaching and therefore being at risk of overtraining syndrome [13, 16, 19]. One study posed the question of whether lower cognitive function places the athlete at greater risk of overtraining rather than overtraining decreasing cognitive function [13]. However, to our knowledge no research has explored this hypothesis, leaving an avenue for future research. Findings from this review suggest that there is a possible correlation between overtraining and cognitive function. Cognitive function tests such as the Stroop test or reaction time tests could be implemented along with questionnaires such as the Multi-Component Training Distress Scale (MTDS) Profile of Mood States (POMS), or Recovery Stress Questionnaire (REST-Q) in athlete monitoring systems to detect early markers for non functional overreaching or overtraining syndrome.

The literature included in this review comprised athletes with an average age of 28 years and most of the studies utilised male participants with females only represented in two of the seven studies. Similar to the studies which included only males [14–17], females’ cognitive function was impaired; however, it is not clear whether either of these two studies including females were adequately powered to make an assessment of the effect of sex on cognitive function in FOR/NFOR athletes [13, 19]. Consideration of why females have not been included in more of the previous research needs to be given. In elite runners, 60% of female athletes recorded overtraining compared to 64% of males [21]. It would therefore be expected that females might show similar symptoms of overtraining as males and consequently it is expected that overtraining would also be associated with impaired cognitive function in females. Participant results from male only studies using the Stroop colour word test showed increases in reaction time after periods of overreaching [15, 16]. These results are similar to those found in the study which used the Stroop test to determine changes in cognitive function in a combined biological sex population and found an increase in mistakes by athletes who had undergone overreaching [13]. Despite these results not being directly comparable due to measuring different variables from the Stroop test they do demonstrate that female athlete cognitive function decreases due to overtraining. Further research should be undertaken in female cohorts to confirm if cognitive function is sensitive to overtraining.

Despite youth athletes also being at risk of overtraining, the studies included in this review only used adult populations [22]. This provides us with little to no indication of how youth athletes’ cognitive function may be impacted by overtraining. Historically, less is known about overtraining in young athletes when compared to adults; however, the psycho-physiological symptoms have been reported to be similar [22, 23]. For example, an English study of athletes aged 11–18 years found that 29% of athletes self-reported being overtrained at least once in their sporting career [22]. Similar results were found in 13–18 year-old swimmers from Greece, Japan, Sweden and the USA with 35% recording overtraining at least once [23]. The prevalence of overtraining was found to be greater in female youth athletes (36%) than in (26%) and was more likely to occur in athletes who competed in individual sports [22]. In youth athletes overtraining may be difficult to characterise based on a decline in performance since performance may decrease in these athletes due to other factors such as schoolwork, exams, or family issues [22]. Research does, however, suggest that symptoms of overtraining in youth athletes are similar to those found in adults. However, youth athletes have reported additional symptoms such as increased conflict with family, coaches, and inability to concentrate [22]. In the literature examining overtraining in youth athletes, it has been found that many athletes who reported being overtrained felt that they were unable to cope with schoolwork and meet the demands which teachers placed on them [22, 24].

Areas for future research on overtraining and cognitive function may include exploring the usefulness of using cognitive tests such as the Stroop test or reaction time tests as a monitoring or diagnostic tool for overtraining. They could be evaluated to determine their usefulness alongside or in place of the subjective wellbeing questionnaires widely used (e.g. REST-Q, POMs, MTDS) to detect early signs of overtraining. It will also be important to explore the effects that overtraining has on both youth’s and older athletes’ cognitive function as there are obvious gaps in the literature surrounding different population groups. Future work should ensure that studies include both male and female participants given the scarcity of research in the female athlete population. This area for further research may be useful in assisting student athletes to manage training and competition schedules alongside academic pursuits, as between 20 and 40% of youth athletes report being overtrained at least once in their careers [22, 23]. Given that there is an expected learning effect caused by impaired cognitive function in FOR athletes, it would be useful to explore the size of the learning effect and if it transfers across into non sport related learnings. Knowledge such as this would be beneficial in assisting athletes who also pursue academic endeavours. Finally, there is a need for research to include a higher percentage of female athletes especially considering that there are similar incidences of females who experience overtraining [21, 22]. Despite the expectation that overtraining affects cognitive function in the same way as it does males, the difference between the biological sexes may be a useful area to explore due to the inherent differences between males and females.

### Limitations

The aim of this review was to investigate the effects of overtraining on cognitive function. However, due to the ethical implications of overtraining potentially causing athletes harm (e.g. performance decrements, injury or illness) many of the included studies used a protocol designed to induce FOR, which is at the low end of the overtraining spectrum. Athletes may not have wanted to participate in these studies due to the possible implications an overtraining protocol may have on their training and consequently performance, as was reported in two of the studies included [15, 16]. Further methodological challenges occur with cognitive function testing in the context of overtraining studies as a learning or practice effect may occur with cognitive function tests if administered too regularly. Another limitation is that these studies included small populations all with similar age groups, with most athletes being over the age of 20 years old and under the age of 30, which may lead to missing the possible effects that overtraining may have on youth and older athletes. Further to this there were only a small number of studies which included female populations. Finally, there is the inherent limitation that the athletes included in the overreaching protocol studies may not have reached a true state of overreaching, with consideration of athletes having different baseline fitness levels not having been taken into account, which potentially impacted on the outcomes of the studies and consequently the impact on cognitive function.

## Conclusion

Cognitive function decreases in response to increases in training load in endurance athletes. This was observed in athletes participating in swimming, running, cycling and triathlon. Cognitive function is a sensitive parameter to overtraining caused by high training volumes. The knowledge gained by this systematic review may be useful for coaches and sport science staff working with endurance athletes. They can look to use cognitive function testing as an early indication to see if athletes are approaching FOR/NFOR given that cognitive function may be a sensitive parameter for the detection of overtraining in athletes.

## Data Availability

The data for this systematic review were not generated by the authors, as the study only included a review of existing literature. No original data were collected or analysed. The sources of the data are provided in the references section of the paper.

## Abbreviations

PRISMA: Preferred Reporting Items for Systematic Reviews and Meta-analysis
MTDS: Multi-component Training Distress Scale
POMS: Profile of Mood States
REST-Q: Recovery Stress Questionnaire
